# Antacid Use and *De Novo* Brain Metastases in Patients with Epidermal Growth Factor Receptor-Mutant Non-Small Cell Lung Cancer Who Were Treated Using First-Line First-Generation Epidermal Growth Factor Receptor Tyrosine Kinase Inhibitors

**DOI:** 10.1371/journal.pone.0149722

**Published:** 2016-02-19

**Authors:** Yu-Mu Chen, Chien-Hao Lai, Huang-Chih Chang, Tung-Ying Chao, Chia-Cheng Tseng, Wen-Feng Fang, Chin-Chou Wang, Yu-Hsiu Chung, Yi-Hsi Wang, Mao-Chang Su, Shih-Feng Liu, Kuo-Tung Huang, Hung-Chen Chen, Ya-Chun Chang, Meng-Chih Lin

**Affiliations:** 1 Division of Pulmonary and Critical Care Medicine, Department of Internal Medicine, Chang Gung Memorial Hospital-Kaohsiung Medical Center, Chang Gung University College of Medicine, Kaohsiung, Taiwan; 2 Department of Respiratory Care, Chang Gung Institute of Technology, Chiayi, Taiwan; H. Lee Moffitt Cancer Center & Research Institute, UNITED STATES

## Abstract

**Background:**

Antacid treatments decrease the serum concentrations of first-generation epidermal growth factor receptor (EGFR)-tyrosine kinase inhibitors (TKIs), although it is unknown whether antacids affect clinical outcomes. As cerebrospinal fluid concentrations of TKIs are much lower than serum concentrations, we hypothesized that this drug-drug interaction might affect the prognosis of patients with *de novo* brain metastases.

**Materials and Methods:**

This retrospective study evaluated 269 patients with EGFR-mutant non-small cell lung cancer (NSCLC) who had been diagnosed between December 2010 and December 2013, and had been treated using first-line first-generation EGFR-TKIs. Among these patients, we identified patients who concurrently used H2 receptor antagonists (H2RAs) and proton pump inhibitors (PPIs) as antacids. Patients who exhibited >30% overlap between the use of TKIs and antacids were considered antacid users.

**Results:**

Fifty-seven patients (57/269, 21.2%) were antacid users, and antacid use did not significantly affect progression-free survival (PFS; no antacids: 11.2 months, H2RAs: 9.4 months, PPIs: 6.7 months; p = 0.234). However, antacid use significantly reduced overall survival (OS; no antacids: 25.0 months, H2RAs: 15.5 months, PPIs: 11.3 months; p = 0.002). Antacid use did not affect PFS for various metastasis sites, although antacid users with *de novo* brain metastases exhibited significantly shorter OS, compared to non-users (11.8 vs. 16.3 months, respectively; p = 0.041). Antacid use did not significantly affect OS in patients with bone, liver, or pleural metastases.

**Conclusion:**

Antacid use reduced OS among patients with EGFR-mutant NSCLC who were treated using first-line first-generation EGFR-TKIs, and especially among patients with *de novo* brain metastases.

## Introduction

The incidence of lung cancer is increasing in Taiwan, and lung cancer is the leading cause of cancer-related deaths worldwide.[1–4] Relatively high incidences of epidermal growth factor receptor (EGFR) mutations have been reported among patients with an Asian lineage, never smokers, and cases of adenocarcinoma.[5–7] Nevertheless, EGFR-tyrosine kinase inhibitors (TKIs) improve progression-free survival (PFS), overall survival (OS), and quality of life outcomes among non-small cell lung cancer (NSCLC) patients harboring EGFR mutation. Furthermore, EGFR-TKIs are less toxic than platinum-based doublet chemotherapy.[8–10]

Antacids decrease the area under the plasma drug concentration-time curve and peak plasma concentration of first-generation EGFR-TKIs by 33–70% among healthy volunteers,[11] although the effects of this drug-drug interaction on the survival outcomes of previous studies remains debatable.[12, 13] Nevertheless, the concentrations of TKIs in the cerebrospinal fluid (CSF) are less than the serum concentrations,[14–17] and EGFR-TKIs are only effective for a portion of patients with brain metastases from NSCLC.[18] Therefore, we hypothesized that patients with *de novo* brain metastases from NSCLC would be more likely to be affected (i.e., experience less tumor control and/or new metastases) by the interaction between antacids and EGFR-TKIs.

## Material and Methods

### Patient and Clinical Characteristics

This retrospective study evaluated patients with NSCLC who were diagnosed between December 2010 and December 2013 at Kaohsiung Chang Gung Memorial Hospital in Taiwan. All patients were subsequently followed-up until June 2015. The inclusion criteria were age of >18 years, histologically or cytologically confirmed advanced-stage NSCLC with *EGFR* mutations, and first-line treatment with first-generation EGFR-TKIs. Patients were excluded if they had previously received any targeted therapy, chemotherapy, or immunotherapy. This study’s design was approved by the institutional review board of Kaohsiung Chang Gung Memorial Hospital, and the requirement for informed consent was waived, due to the retrospective design.

Baseline assessments were performed within 4 weeks of treatment initiation, including clinical characteristics and findings from chest radiography, chest computed tomography, bone scan, and brain magnetic resonance imaging. The clinical characteristics included age, sex, smoking status, Eastern Cooperative Oncology Group (ECOG) performance status (PS), diabetes mellitus, EGFR mutations, and sites and symptoms of distant metastases. We also recorded whether the patient was concomitantly using antacids (proton pump inhibitors [PPIs] or H2 receptor antagonists [H2RAs]) while also receiving TKI treatment, and the duration of concomitant use as a proportion of the TKI-treatment period. Patients who exhibited an overlap of >30% between antacids and TKIs usage days were defined as antacid users. Among patients who used more than one antacid, we only considered the antacid with the greatest overlap.

### *EGFR* Mutation Testing

Tumor specimens were obtained from biopsy samples that were obtained via bronchoscopy, computed tomography-guided biopsy, or surgical procedures. Tumor specimens from pleural effusion cytology were also considered acceptable. The genetic analyses were performed using Scorpion primers and genomic DNA that was extracted from the paraffin-embedded tissues (QIAGEN EGFR RGQ PCR Kit), which was subjected to amplification refractory mutation system-polymerase chain reaction.[19] Deletions in exon 19 and the L858R mutations were defined as “common” mutations, and all other mutations (rare and/or compound) were defined as “uncommon” mutations.[20]

### Evaluating Response to EGFR-TKI Treatment

To evaluate the tumor response, patients underwent chest radiography every 2–4 weeks and chest computed tomography every 2–3 months. Disease status was determined by the attending clinician according to Response Evaluation Criteria in Solid Tumors guidelines (version 1.1).[21] PFS was defined as the period from the first day of EGFR-TKI treatment until disease progression, death before documented progression, or the last visit during the follow-up period. OS was defined as the period from the first day of EGFR-TKI treatment until death, loss to follow-up, or the last follow-up. Patients with various sites of metastases (e.g., brain, bone, liver, and pleura) were further subdivided into antacids users or non-users, and their PFS and OS were compared.

### Statistical Analyses

Statistical analyses were performed using MedCalc software (version 14.10.2) and receiver operating characteristic curves. The PFS and OS analyses were performed using the Kaplan-Meier method and the log-rank test. A Cox proportional hazards regression model was used to evaluate independent factors that affected the survival outcomes. A p-value of <0.05 was considered statistically significant.

## Results

### Patient Characteristics

**Fig 1:**
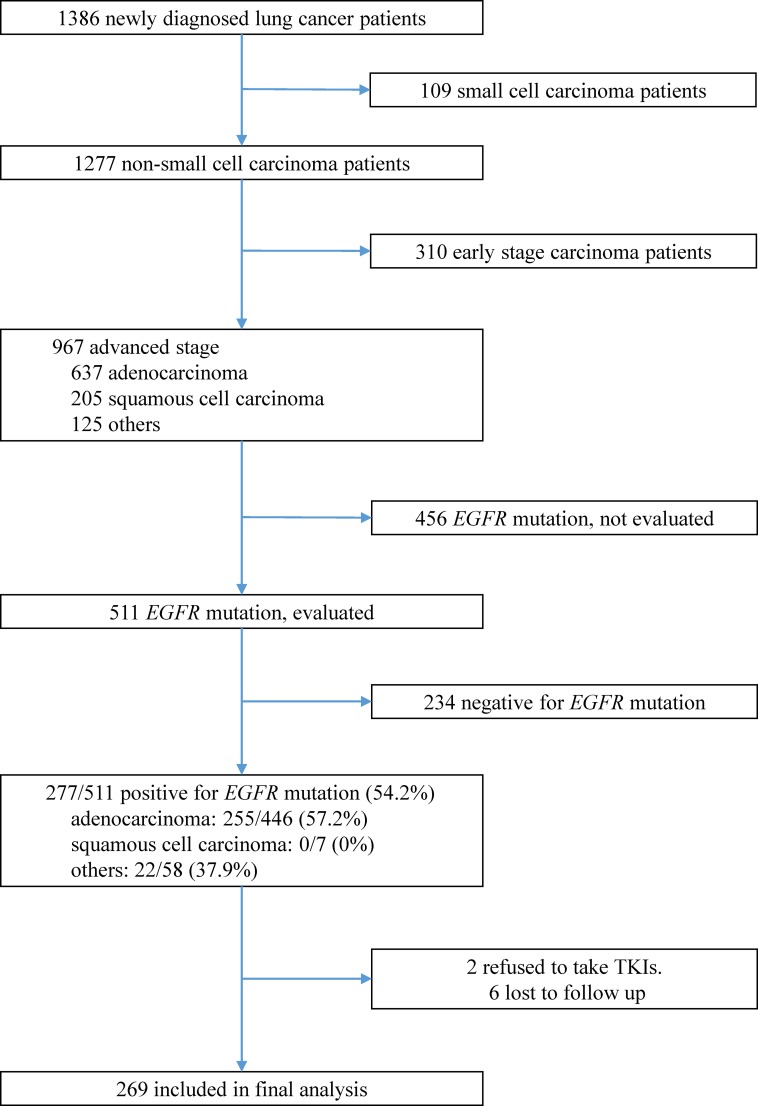
The inclusion, screening, and group assignments for this study. Among 1,386 patients who were diagnosed with non-small-cell lung cancer between January 2011 and January 2014, 269 patients were included in the final analysis.

Among 1,386 patients who were diagnosed with lung cancer between December 2010 and December 2013, we identified 511 patients with advanced NSCLC who were screened for *EGFR* mutations (Fig 1). Among these patients, 277 (57.2%) patients had EGFR-mutant NSCLC. However, 2 patients refused to receive TKI treatment and 6 patients were lost to follow-up; therefore, 269 patients were included in the final analysis. The mean patient age was 65.1 ± 12.3 years, 42.0% (113/269) of the patients were male, 23.8% (64/269) of the patients had *de novo* brain metastases, 44.2% (119/269) of the patients had *de novo* bone metastases, 13.0% (35/269) of the patients had *de novo* liver metastases, and 48.0% (129/269) of the patients had *de novo* pleural metastases (Table 1).

**Table 1:** The clinical characteristics of patients who did and did not use antacids.

	All patients (n = 269)	Used antacids (N = 57, 21.2%)	No antacids (N = 212, 78.8%)	P-value
	n (%)	n (%)	n (%)	
**Clinical characteristics**				
Age, years	65.1 ± 12.3	66.6 ± 14.2	64.6 ± 11.7	0.286
Sex				0.133
Male	113 (42.0)	29 (50.9)	84 (39.6)	
Female	156 (58.0)	28 (49.1)	128 (60.4)	
ECOG performance status				0.052
0	50 (18.6)	3 (5.3)	47 (22.2)	
1	167 (62.1)	41 (71.9)	126 (59.4)	
2	28 (10.4)	6 (10.5)	22 (10.4)	
3	19 (7.1)	5 (8.8)	14 (6.6)	
4	5 (1.9)	2 (3.5)	3 (1.4)	
EGFR mutation				0.890
Common	242 (90.0)	51 (89.5)	191 (90.1)	
Uncommon	27 (10.0)	6 (10.5)	21 (9.9)	
Tumor type				0.466
Adenocarcinoma	247 (91.8)	51 (89.5)	196 (92.5)	
Non-adenocarcinoma	22 (8.2)	6 (10.5)	16 (7.5)	
No. of distant metastasis				0.122
0	31 (11.5)	4 (7.0)	27 (12.7)	
1	133 (49.4)	23 (40.4)	110 (51.9)	
2	62 (23.0)	18 (31.6)	44 (20.8)	
3	32 (11.9)	7 (12.3)	25 (11.8)	
≥4	11 (4.0)	5 (8.8)	6 (2.9)	
Brain metastasis	64 (23.8)	18 (31.6)	46 (21.7)	0.120
Bone metastasis	119 (44.2)	25 (43.9)	94 (44.3)	0.948
Liver metastasis	35 (13.0)	12 (21.1)	23 (10.8)	0.074
Pleura metastasis	129 (48.0)	33 (57.9)	96 (45.3)	0.091
**Adverse events**				
Skin rash	116 (43.1)	19 (33.3)	97 (45.8)	0.102
Diarrhea	33 (12.3)	4 (7.0)	29 (13.7)	0.253
Interstitial lung disease	2 (0.1)	0 (0.0)	2 (0.9)	1.000

ECOG, Eastern Cooperative Oncology Group; EGFR, epidermal growth factor receptor.

At the last follow-up, disease progression was noted in 84.8% (228/269) of the patients, and 48.3% (130/269) of the patients were alive. The median PFS was 10.3 ± 0.7 months, the median OS was 22.0 ± 1.6 months, the partial tumor response rate was 79.2%, and the disease control rate was 88.5% (stable disease was detected in 25 of the 269 patients). The median follow-up was 24.5 months, and the longest follow-up was 47.2 months.

### Antacid Use

Among the 269 patients, 57 (21.2%) patients were considered antacid users (>30% overlap between antacid and TKI treatment). Thirty-nine (14.5%) patients had received H2RAs, and 18 (6.7%) patients had received PPIs. Among the 57 antacid users, 32 (56.1%) patients exhibited an overlap of >80%, 9 (15.8%) patients exhibited an overlap of 51–80%, and 16 (28.1%) patients exhibited an overlap of 31–50%.

### Survival Analysis

**Fig 2:**
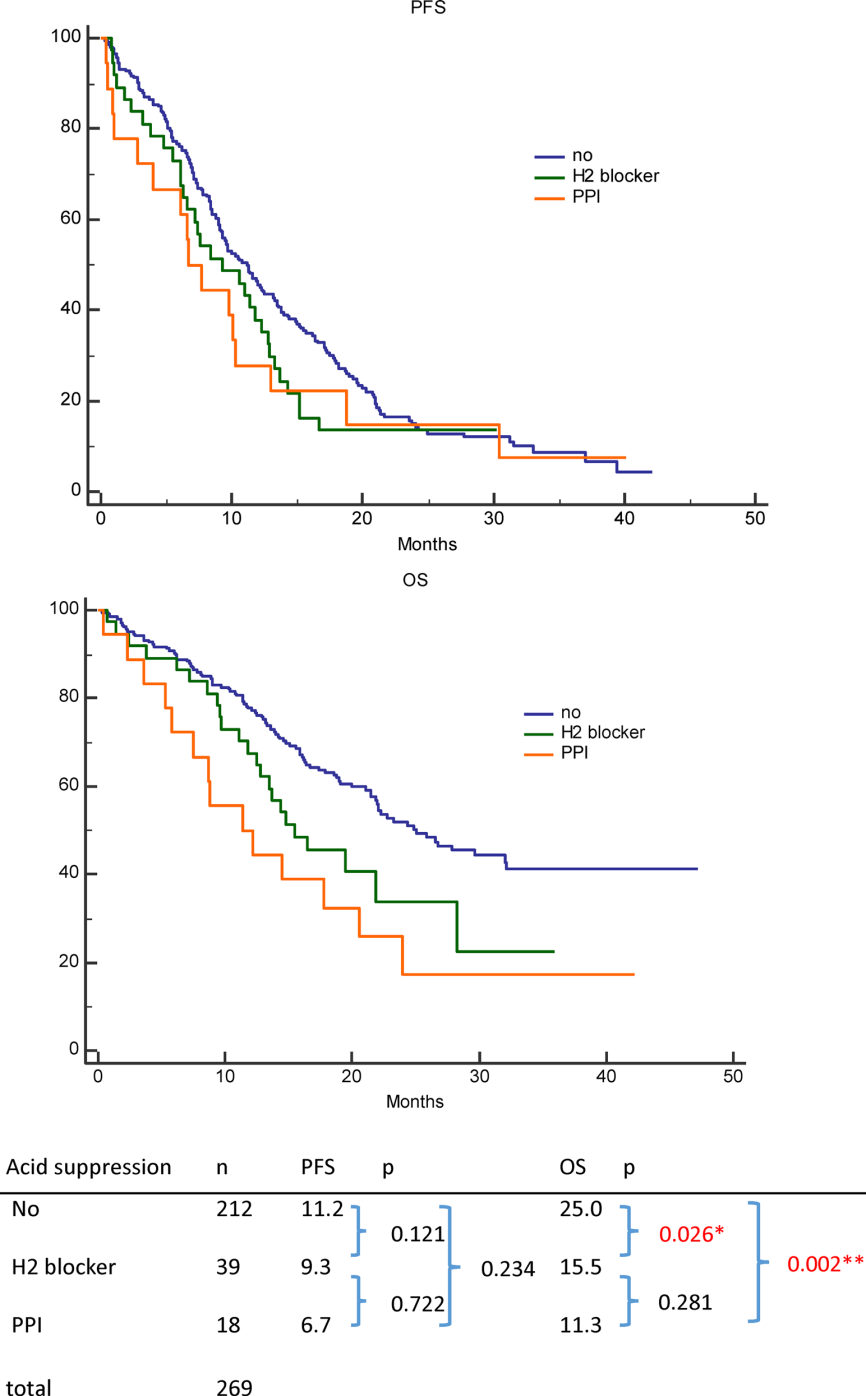
Progression-free survival (PFS) and overall survival (OS) according to antacid use among patients with epidermal growth factor receptor-mutant non-small-cell lung cancer who were treated using first-line tyrosine kinase inhibitors. (top) PFS among patients who were receiving proton pump inhibitors, histamine H2-receptor antagonists, or no antacid. (bottom) OS among patients who were receiving proton pump inhibitors, histamine H2-receptor antagonists, or no antacid.

In the univariable analysis, prolonged PFS was significantly associated with an ECOG PS of ≤2 (p < 0.001), common EGFR mutations (p < 0.001), no brain metastasis (p = 0.001), no bone metastasis (p < 0.001), no liver metastasis (p < 0.001), and no pleural metastasis (p = 0.007) (Table 2). Age, sex, diabetes mellitus, smoking, tumor histology, and antacid use (Fig 2) were not significantly associated with PFS. In the multivariable analysis, prolonged PFS was independently associated with an ECOG PS of ≤2 (p = 0.006), common EGFR mutations (p < 0.001), no bone metastasis (p < 0.001), no liver metastasis (p = 0.005), and no pleural metastasis (p = 0.004) (Table 2).

**Table 2:** Univariable and multivariable analysis of progression-free survival.

	Univariable analysis			Multivariable analysis		
	n	PFS (months)	P-value	Hazard ratio	P-value	95% CI
Age, years			0.204			
>65	134	11.3				
≤65	135	10.0				
Sex			0.702			
Male	113	10.3				
Female	156	10.5				
Diabetes mellitus			0.602			
Yes	51	10.3				
No	218	10.3				
Smoking history			0.683			
Never	182	10.6				
Former / current	87	9.8				
Performance status			<0.001		0.006	
ECOG 0–2	242	11.3		1		
ECOG 3–4	27	2.9		2.16		1.20–2.98
EGFR mutation			<0.001		<0.001	
Common	242	11.3		1		
Uncommon	27	5.1		2.76		1.77–4.32
Tumor type			0.104			
Adenocarcinoma	247	10.6				
Non-adenocarcinoma	22	9.0				
Brain metastasis			0.001		0.180	
Yes	64	7.4		1.25		0.90–1.73
No	205	12.0		1		
Bone metastasis			<0.001		<0.001	
Yes	119	7.8		1.78		1.32–2.40
No	150	13.5		1		
Liver metastasis			<0.001		0.005	
Yes	35	6.7		1.80		1.20–2.71
No	234	11.3		1		
Pleura metastasis			0.007		0.004	
Yes	129	9.0		1.49		1.14–1.94
No	140	11.8		1		
Antacid			0.234			
Proton pump inhibitor	18	6.7				
H2 receptor antagonists	39	9.4				
None	212	11.2				

PFS, progression-free survival; CI, confidential interval; ECOG, Eastern Cooperative Oncology Group; EGFR, epidermal growth factor receptor.

In the univariable analysis, prolonged OS was significantly associated with an ECOG PS of ≤2 (p < 0.001), adenocarcinoma histology (p = 0.007), no brain metastasis (p < 0.001), no bone metastasis (p < 0.001), no liver metastasis (p < 0.001), and concomitant use of antacids (p = 0.002) (Fig 2) (Table 3). Age, sex, diabetes mellitus, smoking, EGFR mutation type, and pleural metastasis were not significantly associated with OS. In the multivariable analysis, prolonged OS was independently associated with an ECOG PS of ≤2 (p = 0.006), no brain metastasis (p = 0.037), no bone metastasis (p = 0.002), no liver metastasis (p = 0.013), and concomitant use of antacids (p = 0.014) (Table 3).

**Table 3:** Univariable and multivariable analysis of overall survival.

	Univariable analysis			Multivariable analysis		
	n	OS (months)	P-value	Hazard ratio	P-value	95% CI
Age, years			0.311			
>65	134	21.8				
≤65	135	22.8				
Sex			0.389			
Male	113	21.9				
Female	156	24.4				
Diabetes mellitus			0.917			
Yes	51	24.0				
No	218	22.0				
Smoking history			0.567			
Never	182	22.0				
Former / current	87	22.8				
Performance status			<0.001		0.008	
ECOG 0–2	242	23.3		1		
ECOG 3–4	27	7.3		2.08		1.22–3.56
EGFR mutation			0.334			
Common	242	22.2				
Uncommon	27	14.0				
Tumor type			0.007		0.087	
Adenocarcinoma	247	25.0		1		
Non-adenocarcinoma	22	15.9		1.54		0.94–2.52
Brain metastasis			<0.001		0.037	
Yes	64	14.3		1.51		1.02–2.23
No	205	25.9		1		
Bone metastasis			<0.001		0.002	
Yes	119	15.8		1.77		1.23–2.54
No	150	32.1		1		
Liver metastasis			<0.001		0.013	
Yes	35	12.2		1.82		1.14–2.92
No	234	24.4		1		
Pleura metastasis			0.115			
Yes	129	21.4				
No	140	24.4				
Antacid			0.002		0.014	
Proton pump inhibitor	18	11.3		2.27		1.26–4.11
H2 receptor antagonists	39	15.5		1.46		0.92–2.33
None	212	25.0		1		

OS: overall survival; CI, confidential interval; ECOG, Eastern Cooperative Oncology Group; EGFR, epidermal growth factor receptor.

### The Effect of Antacids among Patients with Different Metastasis Sites

**Fig 3:**
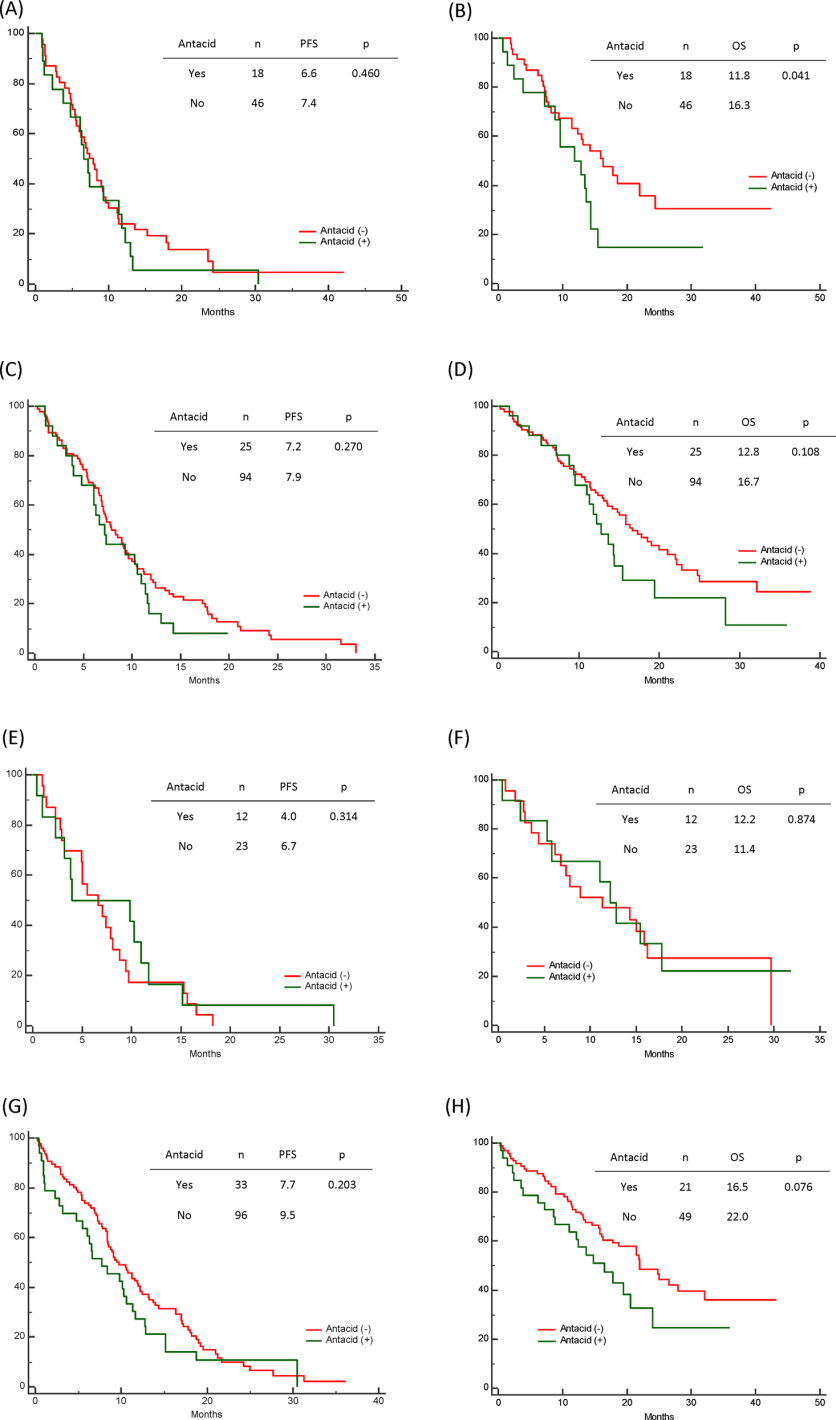
The effect of antacids use and metastasis sites on progression-free survival (PFS) and overall survival (OS) among patients with epidermal growth factor receptor-mutant non-small-cell lung cancer who were treated using first-line tyrosine kinase inhibitors. (A) PFS and (B) OS among patients with brain metastases according to antacid use. (C) PFS and (D) OS among patients with bone metastases according to antacid use. (E) PFS and (F) OS among patients with liver metastases according to antacid use. (G) PFS and (H) OS among patients with pleural metastases according to antacid use.

We also evaluated the effect of antacids among patients with different distant organ metastases (i.e., brain, bone, liver, or pleural metastases). The use of antacids did not significantly affect PFS in this analysis (Fig 3), although antacid users with *de novo* brain metastases exhibited significantly shorter OS (antacid users: 11.8 months, non-users: 16.3 months; p = 0.041). No significant differences in OS were observed when we compared antacid users and non-users among patients with bone, liver, or pleural metastases.

## Discussion

In the present study, we found that 21.0% of our patients with NSCLC exhibited a >30% overlap between their prescriptions for antacids and EGFR-TKIs.

There is controversy regarding the effect of antacids on clinical outcomes among patients with EGFR-mutant NSCLC who are receiving first-generation TKIs. For example, Hilton et al. reported that antacids did not adversely affect PFS and OS among patients who were receiving TKIs in the second line or later line.[13] In contrast, another study reported that the concomitant use of antacids and EGFR-TKIs shortened the PFS and OS among patients with NSCLC (only 4% of patients were receiving first-line TKIs).[12] However, both studies did not examine the patients’ *EGFR* mutation status, and most of the patients were receiving TKIs after receiving a different first-line treatment. Therefore, the present study only included patients who were receiving first-line TKIs, in order to eliminate any confounding that was related to the line of treatment.

In the present study, we found that antacids significantly shortened OS among patients who were treated using first-line EGFR-TKIs, although there was no significant effect on PFS. However, the mechanism for this phenomenon is not clear, and may be multifactorial. One relevant hypothesis is the second hit theory, whereby the drug-drug interaction’s effect becomes enhanced during re-challenge with the TKI. For example, we found that 25% (67/269) of the EGFR-mutant patients were re-challenged with TKIs after second line of chemotherapy. During both the initial and re-challenge treatments, 11 of 67 patients using antacids, and 49 of 67 patients not using antacids; the concordance rate for antacid status at the initial and re-challenge treatments was 89.6% (60/67). Moreover, antacid use during the TKI re-challenge exhibited a trend towards shortening the PFS, although this difference was not statistically significant (antacid users: 1.4 months, non-users: 2.0 months; p = 0.52). Therefore, studies are needed to evaluate whether antacid-related reductions in PFS during each TKI treatment period might significantly affect OS. Another hypothesis is related to a legacy effect for the drug-drug interaction between antacids and TKIs. In this context, we hypothesize that the reduced serum levels of the TKI might not affect control of the main tumor, although it might affect the control of microscopic or gross metastases. The subclinical effect on the distance metastases would not likely affect PFS during the first line of therapy, although it might affect OS beyond the first line. Therefore, we evaluated PFS among the patients who were eligible to receive second-line chemotherapy (n = 123), and found that the PFSs were 2.9 months (n = 24) for antacid users and 4.2 months (n = 99) for non-users (p = 0.159).

Previous studies have reported that the CSF penetration rate of first-generation TKIs ranges from 1% to 3%.[14–17] Therefore, we examined patients with *de novo* brain metastases, and found that the concomitant use of antacids and TKIs significantly shortened their OS. Based on this finding, we hypothesized that the interaction between antacids and TKIs might further reduce the already insufficient levels of TKIs in the CSF of patients with brain metastases. Another interesting issue is whether antacid use might increase the incidence of new brain metastases during TKI treatment in patients without *de novo* brain metastases. Among patients with NSCLC who were receiving platinum-based chemotherapies, CNS metastasis was observed in 7% of patients at the 1-year follow-up,[22] and we also observed that 6.3% (17/269) of our patients exhibited new brain metastases. However, there was no significant difference in the incidences of brain metastases among patients who were and were not using antacids (antacid users: 1/57 [1.8%], non-users: 16/212 [7.5%]; p = 0.111). Therefore, further studies are needed to address this issue.

The present study had several limitations. First, we did not have access to serial data regarding the patients’ serum TKI levels, and cannot confirm that antacid use affected prognosis by reducing TKI levels. Second, our study used a retrospective design with a small patient population, and prospective studies are needed to validate our findings. Nevertheless, to the best of our knowledge, this is the first study to demonstrate that antacid use adversely affected OS among patients with advanced-stage EGFR-mutant NSCLC who were treated using first-line EGFR-TKIs.

## Conclusion

Antacid use reduced OS among patients with EGFR-mutant NSCLC who were treated using first-line first-generation EGFR-TKIs, and this result was especially pronounced among patients with *de novo* brain metastases.
